# Type 2 diabetes drug glitazone may help lowering risk of dementia by 22%

**DOI:** 10.1097/MS9.0000000000000221

**Published:** 2023-03-25

**Authors:** Areeba Fareed, Samia Rohail, Ateeba Kamran

**Affiliations:** Karachi Medical and Dental College, Karachi, Pakistan

*To the Editor*,

Diabetes is a widespread, chronic health issue that affects millions of people worldwide. It denotes either insufficient insulin production or improper insulin use by the organism. The hormone insulin is required by the body to turn food-derived glucose into energy^1^. Type 2 diabetes (T2D) is expected to affect 6.28% (462 million) of the world’s population and 22% of those who are 70 or older^2^. The phrase ‘type 3 diabetes’ was coined to designate a group of people who develop Alzheimer’s disease and dementia likely as a result of diabetes-related damage and degeneration. According to meta-analytic statistics, people with T2D have a 56% higher risk of developing Alzheimer’s disease dementia^3^.

One possible explanation is that hyperglycemia related to T2D can lead to the formation of amyloid beta plaques^4^. Furthermore, insulin resistance in T2DM effectively results in increased oxidative stress that leads to the production of reactive oxidative species that damage intracellular organelles such as the endoplasmic reticulum and mitochondria, resulting in the accumulation and impaired clearance of misfolded proteins^4^.

Recent research has suggested that different T2D treatments have varying dementia incident rates; nonetheless, oral diabetic medicines have been linked to a decreased incidence of dementia^3^. The use of Metformin and Thiazolidinedione was associated with a lower risk of dementia and improved memory and cognitive function^5^,^6^. In contrast, sulfonylureas and insulin were reported to increase the risk of dementia and impaired cognitive function^5^,^6^. Metformin is the first line of treatment for T2D, but Pioglitazone, a Thiazolidinedione, may be used along with that as the second line of treatment as it provides a protective effect on dementia risk among individuals with T2D^5^,^6^.

Glitazones function by focusing on the peroxisome-proliferated activated gamma receptor, which turns on several genes in the body and is crucial for the body’s metabolism of glucose and fat storage^7^. As a result, they can aid in increasing insulin sensitivity in organs like the liver and skeletal muscle, which increases glucose uptake and reduces hepatic output and maintains the functionality of cells that produce insulin^7^.

Patients who used Glitazones for at least a year had a 22% lower risk of developing dementia than those who took Metformin after an average follow-up of nearly 7 years^8^. Glitazones were linked to a 19% decreased risk of Alzheimer’s disease when used with Metformin^7^. Reduced blood sugar levels and maintenance of the pancreas’ capacity to make enough insulin are two advantages of Glitazones^7^. By raising levels of HDL (or ‘good’) cholesterol and lowering levels of triglycerides, Glitazones also assist in lowering blood pressure and enhancing lipid metabolism^7^. Glitazones have a preventive effect, but it becomes more evident with prolonged usage in older patients and obese individuals^8^.

Insulin injection was long anticipated to be a successful dementia treatment since insulin imbalances in the central nervous system have been linked to the onset of neurodegeneration^9^. Dementia treatments should therefore be accessible and effective thanks to drug repositioning and repurposing of diabetes medications^9^.

Diabetes is a recognized risk factor for dementia, and it is anticipated that repositioning and repurposing of antidiabetic medications may be successful dementia treatments^10^. However, for dementia, it is crucial to comprehend the central regulation of glucose metabolism^10^. Antidiabetes medications have been shown to have some pharmacological effects on the brain and blood–brain barrier permeability, although not all of these effects have been identified^10^. A society free from dementia may become a reality if the pharmacological and pharmacokinetic properties of antidiabetic medications are understood and clinical studies based on these properties are carried out in the future.

## Ethical approval

Not applicable.

## Consent

Not applicable.

## Sources of funding

None.

## Author contribution

A.F., S.R., and A.K. contributed to this study.

## Conflicts of interest disclosure

None.

## Research registration unique identifying number (UIN)

1. Name of the registry: NA.
2. Unique identifying number or registration ID: NA.
3. Hyperlink to your specific registration (must be publicly accessible and will be checked): NA.

## Guarantor

Areeba Fareed.

## Provenance and peer review

Not commissioned, externally peer reviewed.
